# Effective communication of public health guidance to emergency department clinicians in the setting of emerging incidents: a qualitative study and framework

**DOI:** 10.1186/s12913-017-2220-5

**Published:** 2017-04-28

**Authors:** Yasmin Khan, Sarah Sanford, Doug Sider, Kieran Moore, Gary Garber, Eileen de Villa, Brian Schwartz

**Affiliations:** 1 Public Health Ontario, 480 University Avenue, Suite 300, Toronto, ON Canada M5G 1V2; 2 Division of Emergency Medicine, Department of Medicine, University of Toronto, Toronto, ON Canada; 3 University Health Network, Toronto, ON Canada; 4 Department of Clinical Epidemiology and Biostatistics, McMaster University, Hamilton, ON Canada; 5 KFLA Public Health, Kingston, ON Canada; 6 Department of Medicine, University of Toronto, Toronto, ON Canada; 7 University of Ottawa, Ottawa, ON Canada; 8 Peel Public Health, Mississauga, ON Canada; 9 Dalla Lana School of Public Health, University of Toronto, Toronto, ON Canada

**Keywords:** Communication, Health systems research, Emergency medicine, Public health, Emergency preparedness, Complex systems

## Abstract

**Background:**

Evidence to inform communication between emergency department clinicians and public health agencies is limited. In the context of diverse, emerging public health incidents, communication is urgent, as emergency department clinicians must implement recommendations to protect themselves and the public. The objectives of this study were to: explore current practices, barriers and facilitators at the local level for communicating public health guidance to emergency department clinicians in emerging public health incidents; and develop a framework that promotes effective communication of public health guidance to clinicians during emerging incidents.

**Methods:**

A qualitative study was conducted using semi-structured interviews with 26 key informants from emergency departments and public health agencies in Ontario, Canada. Data were analyzed inductively and the analytic approach was guided by concepts of complexity theory.

**Results:**

Emergent themes corresponded to challenges and strategies for effective communication of public health guidance. Important challenges related to the coordination of communication across institutions and jurisdictions, and differences in work environments across sectors. Strategies for effective communication were identified as the development of partnerships and collaboration, attention to specific methods of communication used, and the importance of roles and relationship-building prior to an emerging public health incident. Following descriptive analysis, a framework was developed that consists of the following elements: 1) Anticipate; 2) Invest in building relationships and networks; 3) Establish liaison roles and redundancy; 4) Active communication; 5) Consider and respond to the target audience; 6) Leverage networks for coordination; and 7) Acknowledge and address uncertainty. The qualities inherent in local relationships cut across framework elements.

**Conclusions:**

This research indicates that relationships are central to effective communication between public health agencies and emergency department clinicians at the local level. Our framework which is grounded in qualitative evidence focuses on strategies to promote effective communication in the emerging public health incident setting and may be useful in informing practice.

**Electronic supplementary material:**

The online version of this article (doi:10.1186/s12913-017-2220-5) contains supplementary material, which is available to authorized users.

## Background

Evidence to inform communication between clinical settings and public health agencies is limited despite recommendations to improve processes of information exchange across health sectors [1, 2]. This is particularly salient in the context of diverse threats such as emerging infectious diseases, extreme weather and anthropogenic events. In the face of potential health risks, examples of public health guidance for clinical settings include information on high risk populations, personal protective equipment, immunization, laboratory testing and treatment. Guidance from public health agencies involves recommendations on actions that can help prevent disease or mitigate health impacts [3]. The urgency of a public health threat often requires that clinicians implement guidance immediately, to protect themselves and the public [3].

The emergency department (ED) represents the front line of the health system and a key interface within the community. Clinicians working in EDs are at high-risk for exposure to emerging health threats [4–8]. The ED is unique in that clinicians may lack regular office space to receive guidance, operate on shift work schedules, and work across multiple ED environments. Emerging public health incidents (EPHIs) create particular urgency in communication between public health agencies and EDs. EPHIs refer to all-hazards events caused by infectious disease, natural or anthropogenic causes with the potential to overwhelm or otherwise disrupt routine local capacities due to their timing, scale or unpredictability [9, 10]. Communicating in the EPHI context is distinguished from routine communication and aims for rapid knowledge transfer and uptake.

There is a paucity of literature published on communication between public health agencies and EDs in the EPHI context. Published literature on EPHI communication to clinicians in general focuses on technical aspects. For example, the volume and frequency of alerts that are disseminated in evolving public health situations is reported as a challenge for clinicians [11–14]. Another concern is lack of timeliness of guidance in responding to infectious disease threats [13–16]. Focus on methods of communication, such as details of process and infrastructure, is largely limited to considering the technological solutions to barriers, at the expense of examining the specific contexts within which communication occurs. Some evidence indicates that intermediary sources of information, trust and social relationships play an important role in facilitating communication in the EPHI context [17]. The influence of social factors and relationships is an area that would benefit from further study.

This study uses a complexity theory lens to advance understanding of communication between EDs and public health agencies, two sectors within the health system. Complexity as a theoretical approach involves a set of concepts that can be applied to understand properties of systems and is useful in developing management or intervention strategies, such as in health system improvement [18–22]. Further, complexity is a paradigm that has been applied and proven suitable for understanding the disaster context, which renders it a valuable lens for examining EPHIs [20]. Complexity science has been described as three sets of concepts [20]. The first set relates to system characteristics, specifically the elements of the system and how they are influenced and interact dynamically. Complex systems can be viewed as open in relation to their environment, and the environment also influences the system. These ideas integrate with the second set of concepts, pertaining to change in complex systems. Change involving element interactions displays three characteristics: it is non-linear, is affected by history, and it demonstrates self-organization. In non-linear change, inputs may not be proportional to outputs; implying that the system as a ‘whole’ cannot be reduced to its individual parts. This non-linearity in complex systems is also referred to as emergence [20]. Feedback and change result in a history, in that the past influences present system behaviour [20, 23]. Self-organization refers to how new features arise or organize spontaneously from interactions between elements. Agency is the third set of concepts in complexity and links with the idea of adaptation, where complex systems co-evolve over time. The three sets of complexity concepts, namely system characteristics, change and agency can thus be used to illuminate understanding of the multi-stakeholder, multi-jurisdictional health system in which communication between public health agencies and EDs occurs, in the unpredictable context of EPHIs.

This qualitative study addresses an evidence gap by exploring how specific contexts and social factors influence communication processes between public health agencies and ED settings. We employ the theoretical lens of complexity to further understand the specific ways that EPHIs present challenges to communication, along with strategies to address them. Qualitative evidence generated on effective strategies for communication at the local level in EPHIs is synthesized to develop a framework. Frameworks are applied as a useful approach in public health practice [24]; if developed from empiric research, they can promote evidence-informed practice [25–27]. The development of a framework will therefore contribute to a practice-relevant knowledge gap.

Our study objectives were: first, to explore current practices, barriers and facilitators at the local level for communicating public health guidance to ED clinicians for EPHIs; and second, to develop a framework that promotes effective communication of public health guidance to clinicians in EPHIs.

## Methods

### Design and context

Our study focused on the context of a provincial health system in Canada. We conducted semi-structured interviews with key informants to collect data regarding communication of public health guidance to ED clinicians in the province of Ontario. This manuscript’s reporting of adherence to COREQ guidelines for qualitative studies is found as Additional file 1. Ontario is Canada’s most populous province at 13.7 million [28], with a large geographic size (1.1 million km^2^) [29] and varying population density. The southern part of the province borders the Great Lakes and United States, is more urban, and has the highest population density. Northern areas of the province are more sparsely populated with great distances between communities, affecting health system delivery. In Canada, health system organization, funding and delivery is the mandate of individual provinces; public health services for Ontario are the responsibility of 36 municipally and regionally-based public health agencies, known as public health units (PHUs). PHUs are governed by local boards of health, with their boundaries generally corresponding to those of municipal or regional governments. The boundaries of regional health care delivery structures in Ontario, Local Health Integration Networks (LHINs), differ from PHU boundaries. Hospitals and their EDs correspond to LHIN regions, however, hospitals also reside within a PHU for public health services such as disease reporting and outbreak management. EDs within hospitals receive guidance from both provincial and local PHUs.

This study used a purposive sampling approach to ensure representation from both acute care (EDs) and PHUs, and across varying regions in Ontario. The sample consisted of key informants from local PHUs and hospital EDs with extensive knowledge of and experience in communication between public health agencies and EDs. ED clinician administrators included physician and nurse administrators who are the main point of contact between external information sources and front line clinicians. Both physician and nurse administrators were recruited as they play different roles in the communication of public health guidance. Public health physicians/decision-makers consisted of Medical Officers of Health and Associate Medical Officers of Health who were important participants due to their role in the provision of guidance in EPHIs to acute care settings. In addition, we sampled across health region peer groups with the goal of seeking representation from urban, urban-rural and rural health regions in Ontario. Health regions were defined for the study according to Statistics Canada Peer Groups, as regions with similar population density and socio-economic characteristics [30, 31]. We anticipated that there may be important variation between the experiences of participants in different regions. For example, sparsely-populated northern health regions may face challenges with communication related to their remote location, compared with more urban southern Ontario. The purposive sample was initiated by email and augmented by a snowball recruitment method within professional networks [32–34].

The research was subject to review and approval by the Public Health Ontario Ethics Review Board. An initial interview guide was developed based on a literature review, piloted with knowledge users from the field who provided feedback on the relevance and phrasing of questions, and was revised iteratively. The guides used for each participant group (public health and ED) are included as Additional files 2 and 3, respectively. Interviews were conducted by a research coordinator (SS) with doctoral-level training in qualitative methodology, including data collection techniques. Only participants and researchers were present during data collection. Twenty-six interviews were conducted with 14 public health and 12 ED participants. All participants had knowledge of the organization under which the research was conducted; however, no relationship with the interviewer was established prior to study commencement. Research participants were provided with detailed information about the study and the study objectives and potential outputs were discussed with at least one member of the research team prior to data collection. Informed consent was obtained from each research participant prior to their participation in the study. No participants dropped out of the study. The in-depth semi-structured interviews ranged between 50 and 100 min in length and were by phone or in person in a workplace setting, at the preference of participants. Data were collected over a 4 month period from November 2014 to March 2015. During this time, preparedness and planning for potential Ebola virus disease patients was occurring across Ontario, and representations of public health concern and control measures were pervasive both within the public sphere, as well as in healthcare settings.

### Data analysis

Employing an inductive analytic approach, the descriptive and analytic themes were developed directly from the data collected [35, 36]. The constant comparison method [37] was used to move iteratively between emergent themes and empirical data. Interviews were audio-recorded, transcribed verbatim, and researchers performed quality checks on all transcripts. Transcripts were entered into NVivo™ 9 and data were coded using a coding matrix that was subject to ongoing development as the research team became more immersed in the data over time. Two researchers, the principal investigator (YK) and research coordinator (SS), independently coded each transcript. The principal investigator is a practitioner in both public health and emergency medicine; the coordinator is a doctoral-trained qualitative researcher, who was familiar with the subject matter but not a practitioner in the field. Together, they represented both insider (YK) and outsider (SS) positions with respect to the substantive content area. Both researchers were aware of their positions in relation to the fields (public health and acute care) and engaged in ongoing reflection and deliberation on how this framed data analysis. Practices for ensuring quality and transferability in qualitative research were used, such as prolonged engagement with the data and in-depth description of context-related factors [38]. We developed an audit trail that included interview guides, audio recordings, field notes, and other data analysis products [39]. The analytic themes were generated by developing descriptive codes that were grounded in the data, applying these codes to the data (transcripts) and synthesizing codes to develop conceptual analytic themes. Preliminary themes were discussed with the full research team. When consensus was reached, the coding matrix was refined and reapplied to the data. Using an Integrated Knowledge Translation approach which applies principles of knowledge translation to the entire research process, preliminary and final themes were shared through consultations with knowledge users [40].

Complexity theory was used in this study in later stage analysis, after data collection was completed and descriptive themes were developed [20, 25]. We employed complexity theory in iterative analysis between descriptive themes and theoretical concepts. The synthesis of the data involved ongoing reflection and consultation between authors regarding emerging themes, complexity theory and the developing framework.

## Results

As described above, 26 interviews were conducted. In the ED participant group, interviews were conducted with nine physician administrators and three nursing clinician administrators. Our sample included both groups of professionals across the three types of health regions: urban (7 ED, 5 public health); urban-rural (3 ED, 6 public health); and rural (2 ED, 3 public health). Thus, the sample of 26 participants enabled a breadth of perspectives such that thematic saturation was reached and no new themes emerged from the data [41, 42]. Of note, we had an almost 100% response rate from the public health participants that we contacted, but considerably greater difficulty reaching the ED sub-sample. This is likely in part due to the busy nature of the clinical environment, a theme which is discussed in our findings below.

As themes emerged, participants’ descriptions of practice at the local level were categorized broadly as challenges encountered and strategies employed. As such, detailed descriptive analysis explored challenges and strategies as overarching themes with sub-themes within. In this section, we first present themes organized according to challenges and strategies that frame communication between public health agencies and ED settings, with quotes provided for illustration. We then discuss themes in relation to complexity theory. Finally, we present a framework for guiding effective communication between EDs and public health agencies derived from thematic synthesis.

## Communication challenges

### Coordination of communication and information

The first theme encompasses the coordination of information from multiple sources and organizations that emerged as a challenge for communication between EDs and public health agencies. For example, one ED clinician-administrator expressed frustration around inconsistent methods of communication used across PHUs, which was discernible because physicians in their ED worked in other EDs across two PHUs, and were receiving different forms of communication from each PHU. Where one health unit was using an email listserv to distribute information to clinicians, a neighbouring counterpart was resisting using the same method.

*“[W]hy [one health unit] would have a much better communication network with physicians and why when we said we want these outbreaks emailed to us. ‘Oh no, we have to fax them.’ Well why do you have to fax them? ‘Because that is what we have always done. That is how we will be sure they get there.’” (ED participant 01)*

Examining coordination from the public health perspective, a participant attributes a lack of integration for purposes of communication, in part, to the creation of LHINs. LHINs are regional structures for health-care delivery and organization, as described in Methodology. They were established by legislation in 2006 and at the time of this study, did not include PHUs [43]. As described below, the exclusion of PHUs from LHINs has resulted in silos that present challenges for communication.

*“I can tell you that prior to the LHINs being created, we had a [Health Unit] region health care group. So it was community and hospitals. We held regular meetings… and it served as a very good platform for communication during [name of EPHI]… with the creation of LHINs that has caused problems, because you have pan-LHIN or regional kind of groups that have been set up, but they are very silo in nature. They are very sectoral in nature. So …I am not aware that there is any table that has been set up that regularly brings hospital and community, including public health together. So that is a challenge.” (Public health participant 03)*

A related challenge was inconsistency between information sources; for example, varied messages or actions across institutions as a result of evolving knowledge. A participant describes in the following example referring to Ebola, how the situation could have been managed more effectively through communication.

*“I think one of the things that we kind of fell down on at a leadership level was communicating to people, because the lack of information was perceived to reflect a lack of action and a lack of attention, whereas we were in pretty constant daily dialogue with our LHIN leads, the rest of the hospital, [Local Health Unit], the Ministry, and there was nothing new to say. Because no one had figured it out yet.” (ED participant 03)*

As the above examples illustrate, gaps in coordination of communication across sectors of the health system and organizations can lead to confusion and inconsistency, resulting in groups receiving conflicting or limited information.

### Occupational group characteristics and work environment

The theme of the ED work environment was consistently discussed throughout the interviews. The idea that the ED represents a unique setting was pervasive in the data. The ED was understood as a dynamic environment characterized by its fast pace, staff turnover, and 24/7 nature. Some participants stated that staff could be away for days due to scheduling and would be “out of the loop” when they returned, thus contributing to difficulties in disseminating public health guidance effectively. The characterization of the ED context as “hectic” and a shift work-oriented environment was also described as restricting effective public health communication based on existing practices.

*“They have very hectic shifts. They are not there during regular working hours, so you know we will send something from Public Health let’s say updated guidance on Ebola as an example. They come on the night shift. They would have to go wherever it is posted.” (Public health participant 04)*

The difficulties experienced in attending regular hours meetings and maintaining consistent communication with physicians was described.

*“… our agendas are always very tight, so there are a lot of people that want to come and speak at our meetings… But we only meet, we only meet once a month, and typically you know two-thirds of our department would be a good turnout, so not everybody gets to hear it or see it so that is why I think email is probably the better way…” (ED participant 10)*

Several participants described the ED as a microcosm that functions distinct from public health agencies. As one public health participant noted, this microcosm is characterized by complexity and is not well-understood.

*“We don’t really have a good understanding of the systems and chaos dynamics within an emergency department…[H]ow does the emergency department function as an organism that interacts with other systems around it …How are we doing in communicating to the other side, but I don’t think Public Health understands the other side… I think that is the goal, is in understanding the systems and dynamic nature and culture of an emergency room.” (Public health participant 07)*

In summary, challenges for effective communication between EDs and public health agencies were described as resulting from inconsistent or uncoordinated messaging. The distinct nature and complexity of the ED setting creates challenges in terms of ‘regular’ communication channels, suggesting that a more tailored, adaptive approach considering the setting and audience may enhance communication of public health guidance.

## Communication strategies

### Partnerships and collaboration

Partnerships and collaboration were described as invaluable for effective communication. Participants gave examples of where and how this was occurring within institutions (e.g., hospitals), across institutions (e.g., across EDs), and across sectors (e.g., between PHUs and EDs). Processes of collaboration within institutions were characterized as facilitating understanding of public health guidance, and as supporting decision-making. For example, the role of infection control practitioners within hospitals was cited as a key liaison position. When asked about how they make decisions about implementing public health guidance, one ED participant stated:

*“Well it is a team effort [making decisions about whether and how to act on recommendations and guidance]. Once again I work in collaboration with infection control, and I look to them for a lot of guidance around the likelihood of the risk.” (ED participant 03)*

Many public health and ED participants echoed this with similar illustrations of the importance of infection control practitioners in facilitating communication between PHUs and EDs. Networks for collaboration across institutions were also described as important to assist in the interpretation and implementation of public health guidance.

*“So I rely very heavily on…my partner Chiefs in the other institutions to find out what they are doing. Collectively we end up I think coming up with a pretty stable, rational response. If any one of us made a decision on our own, and just tried to do our own thing, we would probably get it wrong, and then we would be doing [umpteen] different things.” (ED participant 03)*

Participants in jurisdictions that have established relationships between PHUs and EDs described strategies for collaboration that facilitate communication and clarification around guidance.

*“This relationship we have with [local public health decision-maker] at public health in our community is spectacular. And it helps us to keep up to date, and you know if we have a question… then [s/he] will send us the current, you know this is what to do, this is the current thing. We disseminate it, and it is done usually within a day…” (ED participant 10)*

In summary, collaboration at multiple levels was described by participants as essential to processes of interpreting information, coordinating decision-making and action, and seeking feedback, which all contribute to effective communication.

### Methods of communication

The specific methods (e.g., email, telephone) by which participants communicate influence communication. Often, participants described using multi-pronged approaches, where they first used one method, and then followed up with another. Both public health and ED participants described using judgment around the method depending on the context, such as the level of uncertainty or urgency.

*“[I communicate] Almost exclusively by email if it is something urgent. One on one if it is very urgent, like the people working today need to know, then I would call them directly, because not everyone checks their email on their way to work, but normally for stuff that is sort of 24, 48 hours, I send it by email.” (ED participant 03)*

Public health participants discussed using direct contact and bi-directional communication practices to follow up and facilitate closing the communication loop. The multi-pronged, active engagement approach also emerged as a preference of ED participants for similar reasons.

*“…emails get sent off and I am sure someone from Public Health feels ‘my responsibility is done’, but in reality it is not a closed loop communication, and things could be missed. So I would hope that in important situations there is the closed loop communication of a phone call or…If something was truly a very big emerging threat to know that things are being acted on.” (ED participant 12)*

Despite limitations of email indicated above, email was the preferred method of communication of public health guidance to front-line staff by ED clinician administrators, who judged it the fastest way to present information to clinicians. Participants described an array of communication methods that they considered necessary for reaching the ED and front-line clinicians. The participant below indicates how in-person meetings between PHU personnel and ED clinicians are useful, if there is perceived anxiety or discomfort about the particular EPHI.

*“Yeah, it really depends on the situation. I guess on what am I perceiving? Do they have the information they need? Are they understanding it? Are we getting tons of questions? You know, expressions of anxiety and discomfort. Just, unease, then I will go, and speak personally. If I have a message I really want to push out and I can get on the agenda, you know then I will go.” (Public health participant 04)*

Some participants welcomed the duplication of messages as a strategy to ensure that public health guidance successfully reached its intended audience.

*“At times when there is lots of information flowing about a certain topic, like Ebola, you will get duplication, but on a serious or something that is high risk, you need to have that duplication, because it is better to have double the information than not.” (ED participant 09)*

While discussion by participants included technical aspects of communication such as email, it was consistently identified that the EPHI setting required a multi-pronged approach that included the ability to reach out, adapt and provide feedback. Direct contact through phone calls, teleconferences, or in-person meetings enabled a degree of flexibility and nuance to foster understanding and uptake of public health guidance.

### Roles, relationships and relationship-building

Liaison roles were described as valuable for facilitating communication between public health agencies and EDs, and were typically described as enhancing direct contact between these settings. Participants suggested that one contact person who is responsible for public health guidance dissemination to EDs and communicating to multiple points within the hospital might facilitate dissemination of information.

*“[M]aybe to have one contact person (within the ED) that is accountable for a time period to disseminate the information and instead of maybe just putting all information to one source, they can do multiple sources. And then information can be disseminated a little faster.” (ED participant 09)*

Several public health participants corroborated the idea that a strategy to enhance communication would be to identify a key contact within the hospital who is responsible for communication of public health guidance from the PHU to the ED. There was a lack of consensus about who should fill this key liaison position, but some participants suggested infection control practitioners (as described above), or nursing staff/management. This indicated that specific points of contact would vary according to jurisdiction and institution, thus requiring adaptability.

The importance of relationship-building was a prominent theme within the data. The value of investing in relationships over time was emphasized and the distinction for the EPHI setting articulated.

*“I think with the effectiveness piece you have to sacrifice efficiency a little bit in that I think you need to build, I think local public health need to build personal relationships with somebody in the emergency room, somebody who is going to be there… who will help you with your knowledge translation piece when it counts. So that is a little different from the base line communications that we send out…” (Public health participant 01)*

As expressed by this participant, strong relationships enable public health agencies to extend the provision of information (“baseline communication”) to translate knowledge and guidance into practice in a particular ED setting. Many participants discussed the importance of having direct contact with people to foster the relationships necessary for effective communication.

*“So that is why I do go to the rounds, and I think it is partly listened to because of what people hear, and in the press, but it is partly listened to in our context, and becomes relevant in the context because of the relationship that has already been built up and I mean lots of people say that the time to build a relationship is not in a crisis.” (Public health participant 01)*

Importantly, the relationships that are described as facilitating communication are also acknowledged as requiring both time and effort in their development. In analyzing the data, a number of qualities emerged characterizing the relationships involved in communication. These qualities were: *trust, respect, responsiveness, transparency, flexibility, and consultation.* These qualities were found throughout the data, including the quotes in this section, and supported the notion that communication at the local level is contextual and rooted in relationships between individuals.

A related idea described by participants is the valuable role of emergency preparedness and planning activities in fostering relationships that can promote effective communication during EPHIs. Existing or previous planning activities relating to other EPHI events contribute to strengthened communication around any EPHI.

*“Yeah, so I think probably one of the most important roles is anticipation. Is recognizing what might happen, and in anticipation having the partnerships in place so that whatever does happen, we have already got the partnerships in place… So I think a big part of our role is being aware and anticipating and being prepared.” (Public health participant 09)*

In summary, a number of strategies were described by participants as promoting effective communication. Qualities of relationships and individuals involved were highlighted. Development of relationships over time was emphasized, with relevant roles for liaison between the two sectors emerging. Data pertaining to communication methods indicated that an adaptive approach to the EPHI context incorporating multiple methods and responsiveness to feedback was beneficial in promoting both communication and knowledge uptake.

## Synthesis and development of framework

We analyzed the themes presented above using the conceptual lens of complexity theory to further understanding of communication and inform the development of a framework. The three sets of concepts for complexity–system characteristics, change and agency–were applied and examined in relation to the data [20]. A framework for effective communication was thus developed that was grounded in empirical data, and informed by engagement with complexity concepts. The framework aimed to elucidate actionable strategies for enhancing effective communication between PHUs and EDs at the local level. The framework elements are described below and displayed in Table 1. The qualities described in the data are exhibited as cutting across all elements. Consistent with complexity, the elements are not intended as distinct or separate, but as inter-linked and overlapping, and may feed back onto each other.

**Table 1:** A framework for effective communication of urgent public health guidance to emergency department clinicians

Framework element	Description	Qualities
Anticipate	Prepare and plan collaboratively in the non-emergency setting to build capacity for effective communication, bridge sectors and jurisdictions, and build relevant networks	Trusted Respected Responsive Transparent Flexible Consultative
Invest in building relationships and networks	Establish, promote and invest energy in relationships, partnerships and networks	
Establish liaison roles and redundancy	Develop and implement liaison roles to effectively transmit timely communication, promote redundancy across communication channels, and provide opportunity for feedback	
Active communication	Actively engage in multiple modes and methods of communication for EPHI information to facilitate timely dissemination, knowledge translation and provide opportunity for feedback	
Consider and respond to the target audience	Consider the unique demands of the target setting and recognize incongruities across settings. Develop accessible and feasible methods to facilitate the exchange of information that accounts for the setting. Provide practice and bottom-line oriented messages, with changes in information emphasized.	
Leverage networks for coordination	Use networks within and across institutions/sectors/jurisdictions to promote coordinated communication action	
Acknowledge and address uncertainty	Understand, acknowledge and respond to the limitations of the message and situation	

The element *Anticipate* refers to collaborative planning and preparedness in the non-emergency setting that provides a mechanism to support effective communication. *Anticipate* includes the development of relationships (extended below) but also includes the structures and processes that may support communication, such as employment of incident management systems, or preparedness activities, such as scanning the local environment for potential risks (e.g., extreme weather or outbreaks). Conceptualizing this element in relation to complexity illustrates the idea that preparedness activities can promote adaptive agency and dynamic change over time [44, 45]. Thus, the system develops its structure and is able to adapt to manage changes in the environment.

*Invest in building relationships and networks* was described recurrently in the data as an actionable strategy that participants found effective. This overlaps with *Anticipate* but is distinguished from general anticipation and preparedness efforts that are included above by a specific focus on social relationships, due to their prominence in the data. Conversely, participants from jurisdictions without opportunities for cross-sectoral planning that promoted relationship development described challenges in knowing key players and thus communicating across sectors during EPHIs. Social relationships are thus a key element in this framework. As with complex systems, communication networks and relationships that develop over time exhibit adaptive agency. Many participants in this study described their communication approaches as characterized by a certain degree of flexibility, which allowed them to adapt to evolving contexts. Furthermore, the process of relationship development involved time investments, and was grounded in local knowledge and experience. This is consistent with our previous discussion of the qualities of complex systems as beyond the reduction of the whole.

The element *establish liaison roles and redundancy* represents an actionable strategy to facilitate effective communication. Role-based channels and redundancy across communication channels enables communication if people change jobs or are not available. In such cases, communication can be facilitated through specific roles such as infection control practitioners or hospital/nursing leadership. This emphasis on roles is supported by Valaitis et al’s work on the importance of role clarity in successful collaboration across public health and primary care settings [26]. This was also essential to the ED setting in which some actors will be affected by shift work cycles, influencing timeliness of communication. The variation in roles across jurisdictions indicates that roles may exhibit self-organization, based on what works in specific settings, and that this may be affected by feedback influencing the system.

Variety in methods of communication was described as valuable and this was developed as the strategy of *active communication* between public health agencies and EDs. Information disseminated in a manner that permitted it to be easily accessed and consulted was valued, whereas communication without closing the loop, or passively sending information as ‘checking the box’ was seen as insufficient for the EPHI context. Complexity sheds light on the effectiveness of this strategy as it promotes adaptation and self-organization, as well as opportunity for feedback. Specific strategies of *active communication* include verbal and direct communication (in contrast with email) which allow opportunity for nuance and clarification. The idea of a multi-pronged and active engagement approach places an emphasis on understanding and knowledge uptake, which is important for EPHIs.

Several action-oriented strategies emerged under the framework element *consider and respond to the target audience*. The ED audience was recognized as distinct, a microcosm and a challenge for public health agencies to understand. Brokering an understanding of clinician and public health agency roles in an EPHI situation was deemed valuable by participants, considering the importance of the ED audience. The hectic nature of the ED and the differences in working hours related to shiftwork highlighted challenges with more routine channels, such as meetings, and support the active approaches introduced above. Further, the unique ED environment supported the notion of tailored and specific approaches for a target audience. Strategies for tailoring communication to the specific ED audience could include practice and action-oriented messages. This element represents an adaptive approach that adjusts communication strategies to correspond with the specific features of the target environment.

*Leverage networks for coordination* exemplifies complexity concepts of self-organization, feedback and non-linearity. For example, neighbouring institutions (e.g., hospitals) or jurisdictions (e.g., PHUs) that are not coordinated in their communication can create confusion for health care workers and the public, if they organize and communicate differently in the uncertain EPHI setting. Since a complex system is open, interacting with and adapting to its environment, it makes sense to consider that EPHIs and health system players are not restricted to jurisdictional boundaries [25]. Tapping into networks across institutions or jurisdictions can therefore serve to enhance communication by promoting solution-oriented thinking and ensuring partners have a consistent message.

*Acknowledge and address uncertainty* is an important element of a framework for the EPHI setting. This overlaps with the above element in that challenges with coordination may result due to self-organization which can occur in evolving situations, emphasizing the need for acknowledgment of uncertainty. An effective strategy noted by participants involved acknowledging when a message is evolving, a work in progress, or based on limited scientific data. This acknowledgement could be a challenge to public health personnel who feel under pressure to have complete knowledge of the situation about which they are communicating in order to ease anxiety and uncertainty. At the same time, there is increasing acceptance of the importance of transparency about uncertainty, in order to build trust via messaging to the public and other parties, such as health professionals [46].

## Discussion

This study is one of the first to our knowledge that explores the social factors in communication in this context. Previous studies have focused largely on the technical aspects of communication, with discussion around technology and resources, with this study addressing a gap in existing literature. It aligns with previous work in disaster preparedness that describes the importance of ‘soft infrastructure’ or social capital in preparedness and community resilience [25]. The findings from this study provide supporting data that within health systems, social capital and relationships are central to effective communication across sectors during EPHIs, and are crucial to preparedness for such events. These findings are differentiated from much of the research on emergency and hazard management, which tends to focus on technocratic strategies at the expense of socially-oriented approaches [47].

This study reflects diverse perspectives on communication between public health agencies and ED clinicians in the context of EPHIs within Ontario, Canada’s most populous province. Since public health services in other provinces and jurisdictions are defined by similar classifications to those in Ontario, the findings from this Ontario-based study may be transferrable to other provinces in Canada, as well as other countries with similar health systems. It is, however, important to note that regional health structures in Ontario (LHINs) do not include public health agencies, a factor that may result in challenges identified in this study that are not as relevant to health authorities that contain public health structures and functions. Findings from this study may inform better collaboration between public health agencies and clinical services, and promote inter-sectoral communication. In examining communication across sectors, this study also contributes to the evidence base by exploring experiences on both sides of communication, the public health sector and the acute care (ED) sector. In-depth analysis of both perspectives enabled an in-depth examination of barriers and facilitators for both groups and their integration into a framework that reflects collective experiences. Further, our application of complexity theory in analysis illuminated understanding of the multi-stakeholder, multi-jurisdictional health system, and the unpredictable nature of emergencies. Consistent with complexity theory, our results and framework indicate that communication in emergencies is not simply day-to-day activities amplified, but benefits from adaptive and innovative approaches that foster agency within the system [20].

The focus on Ebola virus disease preparedness during the period of data collection influenced study findings in that illustrations of challenges and strategies often referred to Ebola preparedness issues and efforts. During this time period, the province had issued a series of Ebola emergency directives that went directly to health-care workers and their institutions, bypassing local PHUs and their established relationships-communications with EDs. Some examples given by participants may have been specific to these provincial paths of information and consequent confusion and uncertainty that characterized this specific period. It may also have led to narrowing in terms of the descriptions provided in narratives; for example, infectious disease threats were largely discussed, with few examples and illustrations of natural or anthropogenic emergencies. Future research might explore in more depth how this framework applies more specifically to “all hazards” threats.

At the outset of the study, we recognized that communication related to our study objectives was closely linked to principles of knowledge translation and knowledge to action [48]. Specifically, communication from public health agencies to clinical settings during EPHIs includes information for raising awareness that often also requires immediate action. Our focus on social context highlights that in order for public health guidance to be taken up in practice, communication processes must be conceptualized as more than the simple provision of information between settings. In this way, our study describes strategies that may also relate to a rapid knowledge to action cycle [48]. The centrality of trusted, responsive, and co-evolved relationships may inform understanding of knowledge to action processes in other contexts.

In addition to the knowledge to action cycle, this study links with emerging research on collaboration for the public health sector. Valaitis et al. describe an ecological framework for the development and maintenance of successful collaborations between public health agencies and primary care settings [26, 49]. In their framework, intrapersonal, interpersonal, organizational and systemic components are described as framing collaboration across sectors [49]. While Valaitis et al. focused on the primary care non-EPHI setting, the findings from our study further develop the idea of the public health agency as a collaborator across sectors, for which relationships and intra/interpersonal factors are highly important and valued.

## Conclusions

This study explored in-depth perspectives on effective communication between public health agencies and EDs, in the setting of EPHIs. Challenges were explored as well as strategies that may be employed. Synthesis of this data using the lens of complexity theory enabled the description of a framework of actionable strategies for promoting effective communication from public health agencies to ED clinicians. The framework and study findings aim to address a knowledge gap and contribute to practice in both sectors as they work to protect the health of health care workers, individual patients and populations. Next steps can include validation of the framework and examining implementation of the framework in practice.

## Additional files

Additional file 1: COREQ Checklist. Completed checklist regarding study adherence to COREQ guidelines. (PDF 273 kb)

Additional file 2: Interview guide for public health decision-makers. Example of data collection instrument. (DOCX 31 kb)

Additional file 3: Interview guide for Emergency Department clinician administrators. Example of data collection instrument. (DOCX 31 kb)

## Abbreviations

ED: Emergency department
EPHI: Emerging public health incident
LHIN: Local Health Integration Network
PHU: Public health unit
