# Supplementary material for: Effective communication of public health guidance to emergency department clinicians in the setting of emerging incidents: a qualitative study and framework
Source: BMC Health Serv Res. 2017 Apr 28;17:312. doi: 10.1186/s12913-017-2220-5 (PMC5410092; doi:10.1186/s12913-017-2220-5)
Supplement: Supplementary file 2 — Interview guide for public health decision-makers. Example of data collection instrument. (DOCX 31 kb) [file 12913_2017_2220_MOESM2_ESM.docx]

**Project Title: Advancing Effective Communication of Public Health Guidance to Emergency Department Clinicians in Ontario in the Setting of Emerging Public Health Incidents (EPHI)**

*Interview Guide for Public Health Decision-Makers*

**A) Participant’s role/background**

1. Can you tell me about your role in addressing Emerging Public Health Incidents (EPHIs)?

2. Can you tell me, more specifically, about your role in the communication of guidance about EPHIs from public health to Emergency Department (ED) clinicians?

**[Probes: special skills, training or experience required for this position?]**

**B) General Questions**

1. Can you describe the ways in which your organization communicates public health guidance/directives about EPHIs to EDs?

2. What challenges exist around communicating public health guidelines, alerts or other information in the EPHI setting?

**[Probes: What kind or procedures are in place? For an outsider, what does this ‘look like’? Who is involved? Could you describe the process? What works well, what doesn’t work so well?]**

**C) Communication features specific to the EPHI Environment**

1. Is communication about EPHIs different or the same as that around other health issues (give examples up front, such as, antibiotic prescribing recommendations or other clinical alerts)? How? Why do you think this is the case?

**[Probes: ‘dynamic’ versus ‘static’ nature of events, differing parties involved in communication, modes of communication; tailoring of general information and guidance on emergencies/incidents to specific events; excessive demands for information from public health communicators]**

2. Do you face any challenges around the specific content of public health communications (guidance, directives, alerts) that you disseminate around EPHIs? If so, please explain.

**[Probes: changing or uncertain content; guidance as inflexible, infeasible or inconsistent; inappropriate for specific context; actionable, formal or informal; resources available for knowledge uptake]**

3. Do you find communication of guidance or information to be consistent across a range of EPHIs (For example, emerging infectious diseases vs extreme weather emergencies vs environmental emergencies like spills)? What do you think are the reasons for possible inconsistencies?

**[Probe: particular kinds of incidents that receive more attention/investment; perception of risk to public or specific groups; certain kinds of EPHIs require different forms of communication]**

4. Are equity and ethical considerations taken into account in developing guidance for EPHIs that is disseminated to ED clinicians? Why or why not?

**[Probe: risk groups and priority decision-making; ethics and reciprocity, duty to care]**

5. Does communication from public health differ at all according to the ‘phase’ of the outbreak or incident? For example, do you ever communicate with EDs post-incident, or during periods of recovery?

**[Probe: absence or presence of communication in a specific phase as helpful or a hindrance; Communication from public health about what worked or did not following an event; appreciation of healthcare workers contribution following an event]**

**D) Pathways of communication, source and audience characteristics**

1. Are there clearly defined pathways (points of contact) with EDs for communication relating to EPHIs? Please describe. Could you speak to any challenges around pathways of communication between public health and emergency departments?

**[Probe: clearly defined, pre-established, roles for communications, formalized processes; specific person who acts as an interface between public health and EDs; collaboration with other health units]**

2. What do you think are key or desirable features/characteristics of those individuals or organizations that are on the receiving end of information from public health?

**[Probes: legitimacy/trust, reliability/consistency, coordination, transparency, previous relationship or partnership, specific professional role; interpersonal characteristics; characteristics of knowledge users, such as busy schedules of ED clinicians]**

3. How is information tailored to meet the needs of different groups (e.g., clinicians or cleaning staff) working within the ED? Are there any challenges around tailoring guidance? Why or why not?

4. How is communication adapted if ED clinicians request more, or different kinds of, information?

**[Probes: feedback mechanisms]**

**E) Means and forms of communication of guidance on EPHIs**

1. How is information (re: public health alerts or guidance) disseminated from public health to EDs in your jurisdiction? Do you find these to be an effective means of communication? Do you have any suggestions for alternative modes of communication? Do you experience any challenges around inconsistencies in modes of communication between organizations?

**[Probe: email, fax, telephone, text messages, printed info sheets, use of electronic health record; possible inconsistencies in communication infrastructure or preferences across organizations or levels of governance; thoughts about social media]**

2. How is information presented? Are there any challenges around how information is presented in terms of form?

**[Probe: form, structure or organization]**

3. Are you aware of any issues around the frequency of communication (alerts, etc.) from public health about EPHIs?

**[Probe: Experiences and preferences with regards to frequency]**

4. Can you describe the (technical) infrastructure in place to communicate with EDs? Would you say that the existing infrastructure is effective and/or efficient?

**[Probes: Barriers/Facilitators, existing technologies, technical issues]**

**F) Attributes and experiences specific to the organization/department**

1. Do you think that your organization experiences specific challenges or opportunities around communication that might differ from other places/jurisdictions/organizations? Please explain. **[Probe: geography, populations served, staffing issues]**

2. How do you know that the guidance that you have communicated to EDs has been implemented or applied? Are there measures in place to ensure that guidance or directives are taken up? Please describe.

**[Probe: Is there any evaluation done, informally or formally; resources and other supports necessary for guidance uptake and/or behaviour change, role of peer support/encouragement/social, cultural or professional norms; level of perceived self-risk regarding specific issues, such as Ebola versus Pandemic Influenza]**

**G) In closing…**

1. Do you have any recommendations for making communication practices from public health to EDs more effective or efficient? Is there anything that you think I might have missed in my questions today?

2. We have discussed many challenges around communication practices, do you have any thoughts about what “works well” (e.g., processes) from your experience? If you were offered a “wish list” of changes (around practice, resources, and so on) that might improve communication from public health to EDs, what would you request?
