# Supplementary material for: Effective communication of public health guidance to emergency department clinicians in the setting of emerging incidents: a qualitative study and framework
Source: BMC Health Serv Res. 2017 Apr 28;17:312. doi: 10.1186/s12913-017-2220-5 (PMC5410092; doi:10.1186/s12913-017-2220-5)
Supplement: Supplementary file 3 — Interview guide for Emergency Department clinician administrators. Example of data collection instrument. (DOCX 31 kb) [file 12913_2017_2220_MOESM3_ESM.docx]

**Project Title: Advancing Effective Communication of Public Health Guidance to Emergency Department Clinicians in Ontario in the Setting of Emerging Public Health Incidents (EPHI)**

*Interview Guide for ED Clinician Administrators*

**A) Participant’s role/background**

1. Can you tell me about your role in addressing Emerging Public Health Incidents (EPHIs)?

2. Can you tell me, more specifically, about your role in the communication of guidance about EPHIs from public health to Emergency Department (ED) clinicians?

**[Probes: special skills, training or experience required for this position?]**

**B) General Questions**

1. Can you describe the ways in which your institution receives public health guidance/directives about EPHIs?

2. What challenges exist around receiving public health guidelines, alerts or other information in the EPHI setting?

**[Probes: What kind or procedures are in place? For an outsider, what does this ‘look like’? Who is involved? Could you describe the process? What works well, what doesn’t work so well?]**

**C) Communication features specific to the EPHI Environment**

1. Is communication about EPHIs different or the same as that around other health issues (give examples up front, such as, antibiotic prescribing recommendations or other clinical alerts)? How? Why do you think this is the case?

**[Probes: ‘dynamic’ versus ‘static’ nature of events, differing parties involved in communication, modes of communication; tailoring of general information and guidance on emergencies/incidents to specific events; excessive demands for information by various parties]**

2. Do you face any challenges around the specific content of public health communications (guidance, directives, alerts) that you receive around EPHIs? If so, please explain. Do you find guidance from public health to be “clinically-relevant” or easily applied in your practice?

**[Probes: changing or uncertain content; guidance as inflexible, infeasible or inconsistent; inappropriate for specific context; actionable, formal or informal; resources available for knowledge uptake]**

3. Do you find communication of guidance or information to be consistent across a range of EPHIs (For example, emerging infectious diseases vs extreme weather emergencies vs environmental emergencies like spills)? What do you think are the reasons for possible inconsistencies?

**[Probe: particular kinds of incidents that receive more attention/investment; perception of risk to public or specific groups; certain kinds of EPHIs require different forms of communication]**

4. Does communication from public health differ at all according to the ‘phase’ of the outbreak or incident? For example, do you ever receive information from public health post-incident, or during periods of recovery?

**[Probe: absence or presence of communication in a specific phase as helpful or a hindrance; Communication from public health about what worked or did not following an event; appreciation of healthcare workers contribution following an event]**

**D) Pathways of communication, source and audience characteristics**

1. From what sources do you receive information or messaging about EPHI alerts/directives? Do you receive information from other institutions or organizations pertaining to EPHIs? Could you speak to any challenges or opportunities around multiple sources of information in such events? How are these handled within your organization?

**[Probes: are networks in place, other colleagues; Preferences regarding the source of communication for EPHIs (national vs provincial vs local vs international); coordination between different sources; competing or differing priorities; role of professional organizations; inconsistent/inaccurate information; tailoring required if from an outside institution; do you use a point of contact between organizations; role of social media or other media sources in communicating PH guidance to clinicians]**

2. What do you think are key or desirable features/characteristics of the source (individual or organizations) of public health information around EPHIs? What features of sources are important or do you consider when you are making decisions about acting on guidance?

**[Probes: legitimacy/trust, reliability/consistency, coordination, transparency, previous relationship or partnership, specific professional role; interpersonal characteristics; characteristics of knowledge users, such as busy schedules of ED clinicians]**

3. Are there clearly defined pathways (points of contact) for communication practices relating to EPHIs, internal to the organization? Externally, for communication from public health? Please describe. Do you have specific written documentation/guidelines in your hospital about communication from public health to the ED/clinical environment? [Request copies of written policies, etc.]

**[Probe: clearly defined, pre-established, roles for communications, formalized processes; specific person who acts as an interface between public health and EDs]**

4. Do you find information from outside your institution useful in applying to the frontline staff in your department? Are there any difficulties involved in receiving information from outside the department? How is information tailored to meet the needs of different groups (e.g., clinicians or cleaning staff) working within the ED? Are there any challenges around tailoring guidance? Why or why not?

5. What do you do if you feel that you have not received the information about EPHIs that you require from public health?

**[Probes: feedback mechanisms in place]**

**E) Means and forms of communication of guidance on EPHIs**1. How is information (re: public health alerts or guidance) received and disseminated in your institution? Do you find these to be an effective means of communication? Do you have any suggestions for alternative modes of communication?

**[Probe: email, fax, telephone, text messages, printed info sheets, use of electronic health record]**

2. How is information presented? Are there any challenges around how information is presented in terms of form?

**[Probe: form, structure or organization]**

3. Are there any issues around the frequency of communication (alerts, etc.) from public health about EPHIs? Do you receive too few or too many?

**[Probe: Experiences and preferences with regards to frequency]**

4. Can you describe the (technical) infrastructure in place to communicate with PH or EDs? Would you say that the existing infrastructure is effective and/or efficient?

**[Probes: Barriers/Facilitators, existing technologies, technical issues; possible inconsistencies in communication infrastructure or preferences across organizations or levels of governance]**

**F) Attributes and experiences specific to the organization/department**

1. Do you think that your institution/department experiences specific challenges or opportunities around communication that might differ from other places/jurisdictions/organizations? Please explain. **[Probe: geography, populations served, staffing issues]**

2. How and why does your institution or department decide to act on certain EPHIs, and not others? Are there measures in place in your institution/department to ensure that guidance or directives are taken up? Please describe. Do you think that the level of perceived self-risk around specific EPHIs contributes to whether or not measures are taken up? Please explain.

**[Probe: Is there any evaluation done, informally or formally; resources and other supports necessary for guidance uptake and/or behaviour change, role of peer support/encouragement/social, cultural or professional norms; level of perceived self-risk regarding specific issues, such as Ebola versus Pandemic Influenza]**

**G) In closing…**

1. Do you have any recommendations for making communication practices from public health to EDs more effective or efficient? Is there anything that you think I might have missed in my questions today?

2. We have discussed many challenges around communication practices, do you have any thoughts about what “works well” (e.g., processes) from your experience? If you were offered a “wish list” of changes (around practice, resources, and so on) that might improve communication from public health to EDs, what would you request?
